# Assessing viral taxonomic composition in benthic marine ecosystems: reliability and efficiency of different bioinformatic tools for viral metagenomic analyses

**DOI:** 10.1038/srep28428

**Published:** 2016-06-22

**Authors:** M. Tangherlini, A. Dell’Anno, L. Zeigler Allen, G. Riccioni, C. Corinaldesi

**Affiliations:** 1 Department of Environmental and Life Sciences, Polytechnic University of Marche, Via Brecce Bianche, 60131 Ancona, Italy; 2 Microbial and Environmental Genomics, J Craig Venter Institute, San Diego, CA, USA

## Abstract

In benthic deep-sea ecosystems, which represent the largest biome on Earth, viruses have a recognised key ecological role, but their diversity is still largely unknown. Identifying the taxonomic composition of viruses is crucial for understanding virus-host interactions, their role in food web functioning and evolutionary processes. Here, we compared the performance of various bioinformatic tools (BLAST, MG-RAST, NBC, VMGAP, MetaVir, VIROME) for analysing the viral taxonomic composition in simulated viromes and viral metagenomes from different benthic deep-sea ecosystems. The analyses of simulated viromes indicate that all the BLAST tools, followed by MetaVir and VMGAP, are more reliable in the affiliation of viral sequences and strains. When analysing the environmental viromes, tBLASTx, MetaVir, VMGAP and VIROME showed a similar efficiency of sequence annotation; however, MetaVir and tBLASTx identified a higher number of viral strains. These latter tools also identified a wider range of viral families than the others, providing a wider view of viral taxonomic diversity in benthic deep-sea ecosystems. Our findings highlight strengths and weaknesses of available bioinformatic tools for investigating the taxonomic diversity of viruses in benthic ecosystems in order to improve our comprehension of viral diversity in the oceans and its relationships with host diversity and ecosystem functioning.

Viruses are the most abundant biological entities in the world oceans and are considered important drivers of ecosystem processes and biogeochemical cycles^1^,^2^,^3^.

Viral assemblage composition is challenging to characterize due to difficulties associated with cultivation of both host and virus, and the lack of a single gene common to all viral genomes, which prevents the use of approaches analogous to ribosomal DNA profiling^4^,^5^,^6^,^7^. Metagenomics, bypassing these limitations, represents the best current approach for analysing the taxonomic composition of natural viral assemblages and their putative functions^4^,^8^,^9^. Indeed, metagenomic analyses have allowed the characterisation of viral assemblages in different marine ecosystems^4^,^8^,^10^,^11^,^12^,^13^,^14^, shedding light on the marine genetic diversity hitherto hidden.

In benthic deep-sea ecosystems, which cover more than 65% of the world surface^3^, viruses have a recognised key ecological role^3^,^15^, and their genetic richness is expected to be very high^16^. However, viral diversity assessments from such environments are underexplored^15^,^17^. Identifying the taxonomic composition of viral assemblages, in the largest ecosystem on Earth, is crucial for estimating global viral diversity, understanding virus-host interactions, their role in food web functioning and evolutionary processes^18^.

A critical step in the evaluation of viral diversity through metagenomic analyses, besides the generation of viromes not contaminated by the presence of cellular organisms^19^,^20^,^21^,^22^, is the use of reliable bioinformatic tools that provide accurate assessments of viral taxonomic information^23^.

BLAST-based approaches have been utilised in earlier studies to investigate viral taxonomic diversity in marine ecosystems (e.g. by comparing nucleic acid sequences to those available in public databases^9^,^10^,^23^). Other bioinformatic tools have been used for the analysis of (viral) metagenomes, such as GAAS (included in MetaVir), which expand upon the tBLASTx algorithm to add the viral genome size to calculations^17^,^23^,^24^,^25^, Metagenomics RAST (MG-RAST^26^), the Viral Informatics Resource for Metagenome Exploration (VIROME^27^), and the Viral MetaGenome Annotation Pipeline (VMGAP, owned by J. Craig Venter’s Institute^28^). Some of these pipelines are based on ORF-finding algorithms to predict potential coding sequences before comparing them with specific protein databases. All these bioinformatic tools, however, are hindered by the limited number of marine viral genomes (e.g. the RefSeq database hosts 5027 viral genomes on a total of 55966 genomes) deposited in public databases and this leads to a small portion of the entire assemblages being taxonomically described^24^,^29^.

In this study, we compared various annotation tools to investigate their performance in the analysis of taxonomic composition of viral assemblages from different benthic deep-sea ecosystems. We conducted a simulated *in silico* analysis, based on reference viral genomes, to identify the most reliable tools for evaluating viral taxonomic diversity through “virus-oriented” (VMGAP, MetaVir, VIROME^22^,^27^,^28^), and “generalist” pipelines (BLAST, MG-RAST, Naïve Bayesian Classifier-NBC^26^,^30^,^31^). These tools were then applied to our benthic deep-sea viromes to evaluate their effectiveness in classifying viral sequences.

Findings from this study highlight strengths and weaknesses of available bioinformatic tools for investigating the taxonomic diversity of environmental viromes, and for exploring viral assemblage composition in benthic deep-sea ecosystems.

## Results

### Identification of viral sequences and strains in simulated viromes

**Figure 1:**
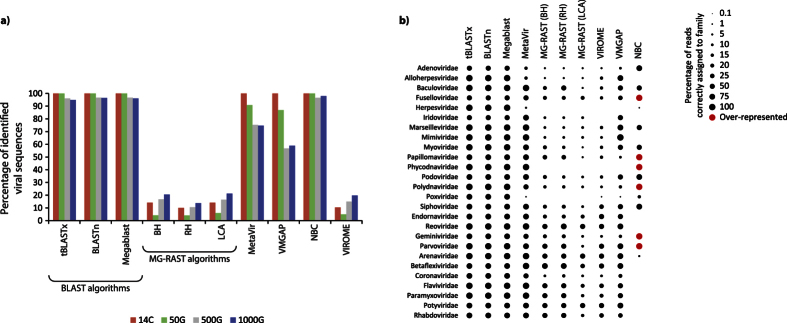
(**a**) Percentage of viral sequences identified in the simulated viromes characterized by a different number of genomes by the different bioinformatic tools. 14C: 14 genomes of *Circoviridae*; 50G: randomly-sampled genomes; 500G: 500 randomly-sampled genomes; 1000G: 1000 randomly-sampled genomes; (**b**) Percentage of viral sequences correctly identified in the simulated viromes for each viral family. Red dots indicate overrepresented viral families (false positives).

All BLAST algorithms (i.e. BLASTn, Megablast and tBLASTx) and the NBC algorithm were able to identify a very high percentage of sequences as viral (up to 100%; Fig. 1a) in the four simulated viromes. MetaVir and VMGAP had a very high efficiency in identifying viral sequences in the 14C sample but it decreased with increasing viral diversity (down to ca. 57–59% in the 500G and 1000G samples). The VIROME pipeline and MG-RAST algorithms Best Hit (BH), Lowest Common Ancestor (LCA) and Representative Hits (RH) classified as viral only less than ca. 20% of the sequences submitted.

In the simulated dataset with 1000 viral genomes, the BLAST algorithms were able to correctly assign almost all sequences to each family, similarly to MetaVir which, however, had a lower resolution in the identification of *Poxviridae*, *Herpesviridae*, *Adenoviridae* and *Alloherpesviridae* (Fig. 1b). In general, VMGAP showed a lower efficiency than MetaVir and failed to identify *Herpesviridae* and *Phycodnaviridae*. MG-RAST’s algorithms and VIROME identified a low percentage of sequences affiliated with eukaryotic viral families (e.g., *Adenoviridae*, *Alloherpesviridae*, *Mimiviridae*, *Coronaviridae*) and were not able to identify *Phycodnaviridae*, *Poxviridae* and *Herpesviridae*. NBC failed in the identification of most of the families affiliated with RNA viruses and provided an overrepresentation of different families (e.g., more than 600% for *Phycodnaviridae*).

**Figure 2:**
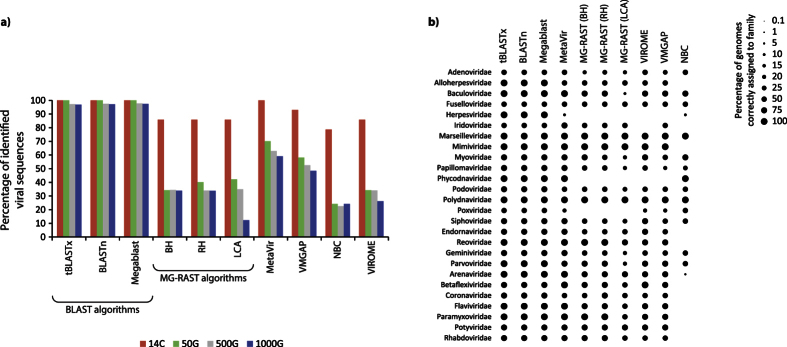
(**a**) Percentage of viral strains correctly identified in the simulated viromes characterized by a different number of genomes by the different bioinformatic tools. 14C: 14 genomes of *Circoviridae*; 50G: randomly-sampled genomes; 500G: 500 randomly-sampled genomes; 1000G: 1000 randomly-sampled genomes; (**b**) Percentage of viral strains correctly identified in the simulated viromes for each viral family.

When viral genomes identified in each sample were counted, we found that BLAST algorithms were able to correctly identify a very high fraction (nearly 100%) of the viral strains used in all samples (Fig. 2a). Among the pipelines, MetaVir and VMGAP were able to correctly identify almost all strains in the 14C sample, but their efficiency declined with increasing number of genomes used for the simulation. MG-RAST, VIROME and NBC provided quite similar results, with the highest fraction of strains correctly identified in the 14C sample; their efficiency, in terms of correct identification of viral genomes, strongly decreased with increasing number of viral genomes used for the simulation (down to 12% for the 1000G sample using LCA algorithm).

In the simulated dataset with 1000 viral genomes, MetaVir identified a lower percentage of viral strains belonging to *Herpesviridae* and *Poxviridae* compared with BLAST algorithms (Fig. 2b). VMGAP and VIROME had a lower resolution power in identifying different viral families and completely failed to identify other families, including *Herpesviridae* and *Phycodnaviridae*. MG-RAST generally showed a low efficiency in the assignment of strains to viral families and was not able to identify three viral families (*Herpesviridae*, *Poxviridae* and *Phycodnaviridae*).

### Identification of viral sequences and strains in environmental viromes

**Figure 3:**
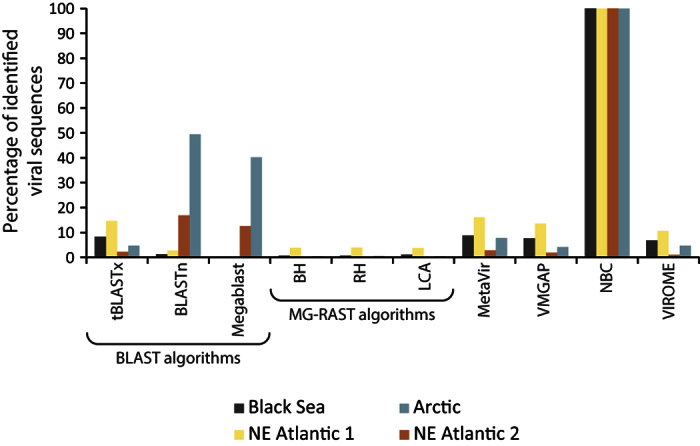
Percentage of viral sequences identified in the viromes generated from deep-sea sediments (Black Sea, NE Atlantic and Arctic Oceans) by the different bioinformatic tools.

**Figure 4:**
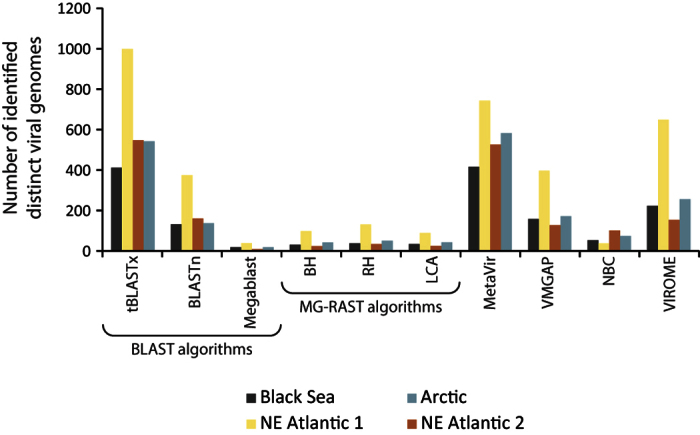
Number of viral strains identified in the viromes generated from deep-sea sediments (Black Sea, NE Atlantic and Arctic Oceans) by the different bioinformatic tools.

The NBC tool appeared to be able to identify almost all sequences as viral in all of the viromes investigated (more than 99%). In contrast, the other tools were able to classify less than 50% of the sequences as viral (Fig. 3). MG-RAST’s algorithms identified a very low number of sequences (0.12% to 3.81%) as viral. TBLASTx classified as viral ca. 8% and 15% of the sequences in the Black Sea and NE Atlantic 1 samples, respectively, while BLASTn and Megablast found more matches to the viral sequence database in the NE Atlantic 2 and Arctic samples. MetaVir, VMGAP and VIROME classified as viral from ca. 1–3% to 11–16% of the sequences (in the NE Atlantic 2 and 1 samples, respectively). The analysis of viral strains in environmental viromes showed that tBLASTx and MetaVir identified the highest number of viral strains (ranges: 405–983 and 409–731, respectively) followed by VMGAP (125–390), BLASTn (157–368) and VIROME (151–639). Conversely, Megablast and MG-RAST’s algorithms identified the lowest number of strains in all samples (Fig. 4).

**Figure 5:**
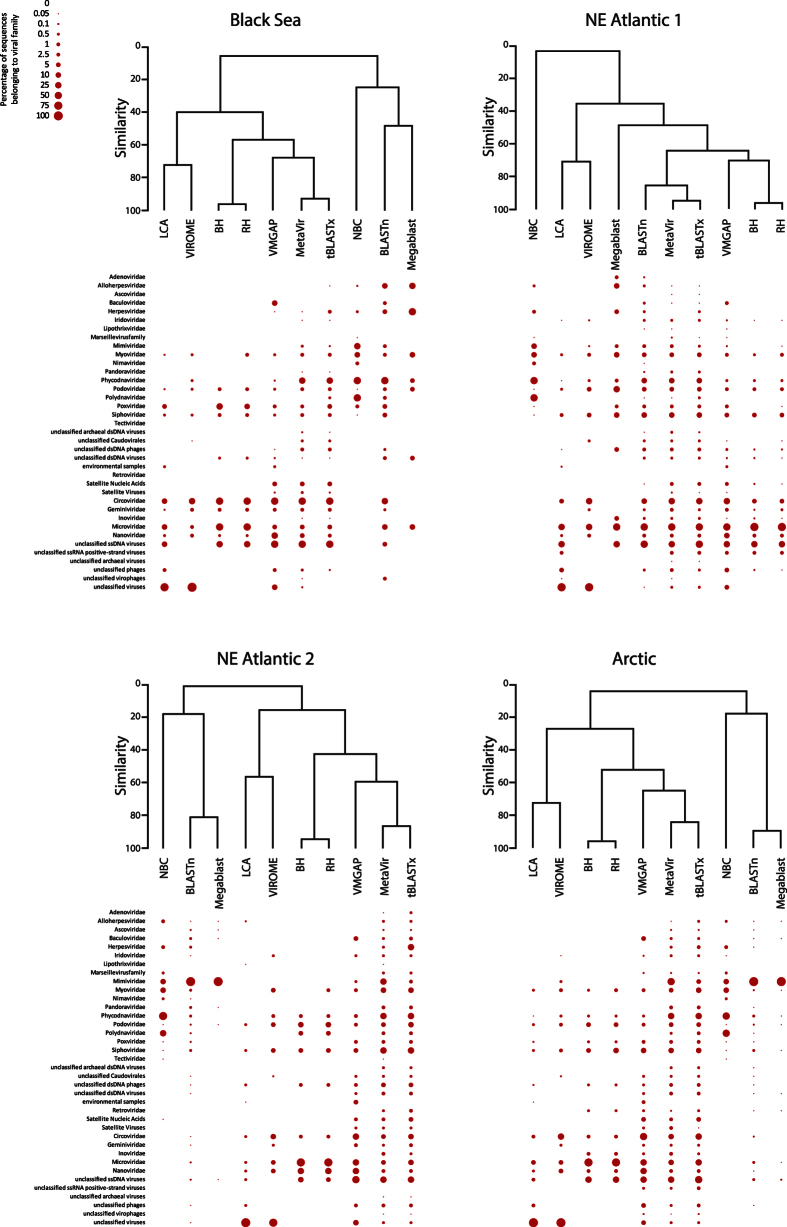
Viral assemblage composition (as contribution of sequences belonging to each viral family) obtained by using the different bioinformatic tools and the relative results of clustering analysis.

**Figure 6:**
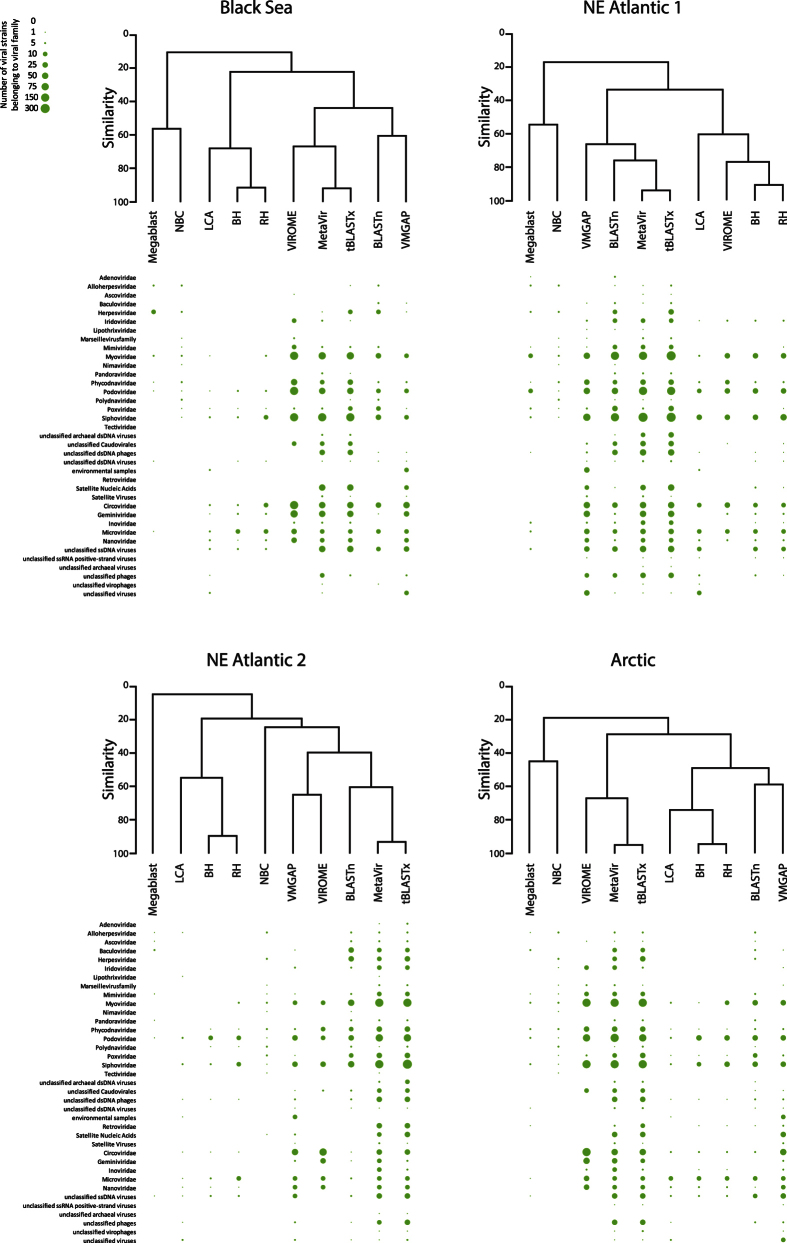
Viral assemblage composition (as contribution of viral strains belonging to each viral family) obtained by using the different bioinformatic tools and the relative results of clustering analysis.

Viral assemblage compositions indicate that NBC failed to identify ssDNA and RNA viruses in all deep-sea viromes, and assigned a much higher fraction of sequences to *Phycodnaviridae* and *Polydnaviridae* than the other tools (Fig. 5). Indeed, NBC showed a very low similarity with every other bioinformatic tool (Fig. 6). Among MG-RAST’s algorithms, BH and RH identified a large fraction of sequences belonging to *Microviridae* family and clustered together with a very high similarity in all environmental viromes (>90%). Conversely, the LCA algorithm assigned a high portion of the viral sequences to the “unclassified viruses” class and clustered with VIROME. Among the BLAST’s algorithms, BLASTn and Megablast generally showed a similar output, identifying a higher fraction of *Mimiviridae* sequences in the Arctic and NE Atlantic 2 viromes than the other tools. However, tBLASTx showed a very high similarity (>90%) with the MetaVir output. MetaVir, tBLASTx, VMGAP and VIROME assigned the sequences to the highest number of viral families. VMGAP generally clustered with tBLASTx and MetaVir, except in the NE Atlantic 1 virome.

The analysis of the number of viral strains affiliated with each viral family indicated that NBC generally clustered with Megablast, showing a very low similarity (<20%) with the other tools considered. BLASTn and tBLASTx, again, generally clustered independently (Fig. 6). In particular, the output of tBLASTx was very similar to that of MetaVir in all samples analysed and resulted in the identification of a high number of strains belonging to Caudovirales (*Myoviridae*, *Siphoviridae* and *Podoviridae*), unclassified archaeal dsDNA viruses and unclassified dsDNA phages than the other tools. Viral assemblage compositions obtained from VMGAP and VIROME clustered differently depending on the virome considered. All outputs of MG-RAST’s algorithms clustered together; however, the similarity between the viral assemblage compositions was higher when BH and RH were considered (>80%).

Results of the analysis carried out on the simulated sequence datasets combined with one of the environmental viromes revealed that the presence of environmental sequences did not influence the identification of simulated genomes (Figure S1). In addition, results of the cluster analysis conducted on the viral assemblage composition of the environmental viromes obtained by contig assembling were consistent with those obtained by analyzing the unassembled sequences (Figure S2).

## Discussion

The advent of metagenomics has represented an unprecedented opportunity for characterizing the viral diversity in marine ecosystems^4^,^29^,^32^. Nevertheless, there are still several obstacles to overcome for capturing the actual viral diversity in such ecosystems, including: i) the limited number of known viral sequences and genomes in public databases^29^, ii) the recovery of an amount of viruses and their DNA sufficient for sequencing, particularly from complex matrices such as marine sediments^33^, and iii) the efficiency of sequencing platforms and bioinformatic tools employed to analyse the viromes.

In the present study, we compared the performance of available bioinformatic tools in analysing the taxonomic composition of viral assemblages in simulated datasets and natural deep-sea sediments. The latter represents the largest biome on Earth and the major repository of viruses and viral infections^3^, and where the composition of assemblages is expected to be complex and highly diversified.

Among the bioinformatic tools used, NBC and BLAST’s algorithms showed the highest number of sequences affiliated independent from the complexity (as number of viral genomes and of viral families used to generate each library) of simulated viromes. However, while the BLAST algorithms were also very efficient in correctly identifying viral strains in the different simulated metagenomes, NBC provided much less effective results as most of the viral groups were completely absent from its annotation results (especially families of RNA viruses). This might be due to: i) the compositional-based approach used by NBC, which is different from the similarity-based strategy used by the other investigated pipelines and algorithms, which also allow setting statistical parameters to assess the significance of the similarity (i.e. E-value), and ii) the low efficiency of the compositional-based approach such as that used by NBC on short sequences (<1 Kb^34^). Moreover, NBC provides by default an affiliation for every input viral sequence, even when a reference database which does not contain any viral genome (e.g., exclusively composed of bacterial genomes) is used (see Supplementary results). All these methodological aspects could severely affect the assessment of viral taxonomic diversity in environmental samples.

Comparing the performance of MetaVir, VMGAP, VIROME and MG-RAST, we observed that all these tools showed a decreasing efficiency in identifying viral sequences and strains with increasing complexity of the viromes considered. However, MetaVir and VMGAP had a higher sensitivity than VIROME and MG-RAST, especially for viral families infecting eukaryotes. MG-RAST and VIROME, indeed, were unable to identify strains belonging to some viral families, including *Herpesviridae* and *Phycodnaviridae*. Since we used the same RefSeq database as a reference for MG-RAST, MetaVir and the BLAST’s algorithms, we excluded that the low effectiveness of MG-RAST can be due to the selection of the reference database. MG-RAST, VIROME and VMGAP equally rely on ORF-finding algorithms developed for prokaryotic gene finding^26^,^27^,^28^ that identify ORFs before their taxonomic annotation. However, both VMGAP and VIROME exploit MetaGene Annotator to find ORFs^28^, which has been shown to have a higher specificity than the software used by MG-RAST, FragGeneScan^35^. In addition, VMGAP also integrates a six-frame translator to MetaGene Annotator and this additional step, capturing all true open reading frames^36^, could be the reason of its higher annotation performance than MG-RAST and VIROME.

Overall, the results of the analyses carried out on simulated viromes indicate that BLAST tools, followed by the pipeline MetaVir and VMGAP, are more reliable in the assignment of viral sequences when compared with other bioinformatic tools. Such results are not influenced by the use of a simulated metagenomic library, since the performance in identifying viral sequences and strains did not change when environmental and simulated viromes were combined.

Bioinformatic analyses of environmental viral metagenomes revealed that NBC provided a much higher number of sequences affiliated (ca. 100%) than the other tools for all viromes investigated. However, as revealed from the analysis of simulated viromes, NBC identified a very low number of viral strains and misassigned a large fraction of sequences and strains (e.g., *Phycodnaviridae*, *Polydnaviridae*), thus indicating that such a tool is not effective to analyse the diversity of natural viral assemblages.

MG-RAST’s algorithms showed a very limited capability to identify viral sequences and strains in all environmental viromes investigated, as obtained for simulated metagenomes. In particular, we confirmed that several viral families, including viruses infecting eukaryotes, were neglected in the environmental viromes. MetaVir, VMGAP and VIROME showed a similar number of sequences affiliated for all environmental viromes, but the number of viral strains identified by MetaVir was higher.

The BLAST algorithms provided very reliable and consistent results among the simulated viral metagenomes, but not when analysing the environmental viromes. Indeed, the annotation of viral sequences and strains was very different when the different BLAST algorithms were applied to the same virome, as also revealed by cluster analysis. These differences can be due to the higher stringency of Megablast compared to BLASTn and tBLASTx, which results in a very low efficiency in the identification of environmental viral strains not strictly related to viral genomes in the reference database. Conversely, tBLASTx enables the identification of more distant relationships between sequences because it also performs a six-frame translation of the query sequences to proteins before the comparison with reference database^31^. Additionally, tBLASTx provided more similar results to those obtained by using MetaVir when applied to environmental viromes (both in the identification of viral sequences and strains, for each virome analysed; >90%) than by using the other BLAST’s algorithms. Such results, based on the analysis of unassembled sequences, were not dependent upon the bioinformatic strategy applied, as we obtained similar results even when assembled contigs were used (Figure S2).

Metavir and tBLASTx also identified a wider range of viral families than all the other tools in environmental viromes, with a better resolution power especially towards strains belonging to Caudovirales (*Myoviridae*, *Siphoviridae* and *Podoviridae*), *Phycodnaviridae*, unclassified archaeal dsDNA viruses and unclassified dsDNA phages. A better resolution power towards viral sequences belonging to Caudovirales and *Phycodnaviridae* was also consistently observed in simulated datasets.

The presence of viral groups infecting eukaryotes such as *Phycodnaviridae* and *Circoviridae* has been identified in all environmental viromes. Since sequences affiliated with viruses infecting eukaryotes were correctly identified in simulated viromes by using MetaVir and the BLAST algorithms, we suggest that the strains identified in the environmental viromes can actually be related to viruses infecting eukaryotes. Viruses affiliated with *Phycodnaviridae* (i.e., infecting algae) could be supplied to the deep-sea floor through particle sinking from the photic zone, whereas the presence of *Circoviridae* could be also related to metazoan hosts (e.g., crustaceans^37^) inhabiting benthic deep-sea ecosystems.

Overall, results obtained here provide evidence that the bioinformatic tools selected for the analysis of environmental viral metagenomes can strongly influence the view of viral diversity in natural ecosystems. Our findings also reveal that tBLASTx and MetaVir are the most suitable tools for the analysis of viral assemblage composition in deep-sea ecosystems. As such, they should be preferred for exploring the diversity of natural viral assemblages and for a better understanding of their relationships with host diversity and ecosystem functioning.

## Methods

### Study areas and sample collection

Undisturbed sediment samples were collected by using a multiple corer in four deep-sea sites at depths ranging from 1970 m to 5500 m. One sampling site was located in the Black Sea at 1970 m depth (42°59′ 54.204″ N, 31°30′ 58.644″ E, hereafter defined Black Sea), two sites in the NE Atlantic Ocean along the Portuguese Margin (39°30′ 24.18″ N, 9°50′ 0.604″ E at 3400 m depth and 41°43′ 51.2394″ N, 10°40′ 56.568″ E at 3000 m depth, hereafter defined NE Atlantic 1 and NE Atlantic 2 respectively), and one site in the Arctic Ocean at 5500 m depth (79°8′ 0.5994″ N, 2°50′ 32.2794″ E, hereafter defined Arctic). After retrieval, sediment samples of the top 1 cm were collected using a sterile spatula and stored at −80 °C until laboratory analyses. Anoxic sediment samples collected in the Black Sea were treated (and subsequently analyzed) under strictly anaerobic conditions (N_2_ atmosphere).

### Recovery of viral particles from sediments

Uncontaminated viral DNA suitable for sequencing analyses was recovered from sediment samples, after isolation of viral particles through a physical-chemical treatment to dislodge viruses from the sedimentary matrix^38^,^39^,^40^, with some modifications. Fifty grams of sediment were diluted with 50 ml of autoclaved virus-free seawater (pre-filtered through 0.02-μm-pore-size filters) and homogenized by stirring for 10 minutes. The slurry was divided into aliquots of 2 ml in 50 sterile tubes and each aliquot was added with 8 ml virus-free seawater (10 ml final volume) containing tetrasodium pyrophosphate (5 mM final concentration).

Samples were incubated for 15 minutes in the dark and then sonicated (40 Khz) in an ultrasonic bath for 3 times of 1 minute each, with 30 seconds of manual shaking after each cycle (Bransonic Branson 3510). The samples were then centrifuged at 800 × g for 10 minutes to reduce the interference due to suspended particles, and the supernatants were recovered. The sediment was homogenized again with virus-free seawater and centrifuged (800 × g for 10 minutes). This step was repeated two times more. All of the supernatants (final volume ca. 600 ml) were combined and filtered through 0.2 μm pore size filters (Millipore). The supernatants were treated with DNases (5U ml^−1^) to remove extracellular DNA. To check for the potential prokaryotic contamination and to assess the extraction efficiency of viral particles from the sediments, aliquots of 0.2 μm pre-filtered samples were diluted with virus-free seawater, stained with SYBR Gold and analyzed by epifluorescence microscopy^40^.

### Viral DNA extraction, amplification and sequencing

Viruses were concentrated onto 0.02 μm filters by vacuum filtration. Each filter was added with virus-free milliQ water and sonicated (three times for 1 minute, with 30 second of manual shaking between each cycle) to detach viral particles. Viral DNA was extracted and purified according to Sambrook *et al*.^41^ with some modifications. Briefly, each filter was incubated at 56 °C for one hour with 20 mM EDTA, 10% SDS and 50 μg ml^−1^ proteinase K. Viral DNA was purified through two subsequent phenol-chloroform treatment steps followed by isopropanol precipitation. Viral DNA was quantified fluorometrically (NanoDrop 3300) using SYBR Gold^19^. To reach the amount of DNA required for pyrosequencing, replicate samples of viral DNA (n = 3) were amplified by using GenomiPhi V2 kit (GE Healthcare). Pooled replicates were purified using Wizard PCR and Gel Clean-up kit (Promega). Before pyrosequencing, potential contamination due to prokaryotic and eukaryotic DNA was checked in the viral DNA samples by PCR targeting 16S and 18S rRNA genes and gel electrophoresis analysis. All of the samples passed the quality check.

Viral DNA libraries were prepared and sequenced at the Broad Institute of MIT and Harvard using a 454 FLX Titanium platform. Sequencing artifacts and low-quality sequences were removed with the PRINSEQ software^42^ and resulting high-quality reads were analysed by the MG-RAST v3 server^26^.

### Generation of simulated virome datasets

Four simulated viral metagenomes were created *in silico* by using the shotgun sequence simulator Grinder^43^ with the following parameters: 450 bp of sequence length, 50 bp of deviation, 454 error mode. The simulated metagenomes, containing 10^5^ sequences each, were generated respectively with 1000, 500 and 50 randomly-selected viral genomes (here defined 1000G, 500G and 50G) from the RefSeq database, without including potential non-viral contaminants (commonly found in environmental viromes; 21). The different number of viral genomes belonging to different strains was selected in order to evaluate the influence of different levels of virome complexity on the annotation efficiency of the bioinformatic tools tested.

Another simulated virome containing 50000 sequences was generated with the same parameters used for the other simulated viromes, with 14 *Circoviridae* genomes (here defined 14C). Genomes of the *Circoviridae* family were selected because they share two hallmark genes^44^ exclusively belonging to such viruses (e.g., without homologues in prokaryotic and eukaryotic genomes), thus representing an additional test for evaluating the reliability of the pipelines to correctly identify viral sequences.

To test for the efficiency of NBC in sequence assignment, we generated two additional databases (see Supplementary Information for details).

Finally, to assess whether the results of the simulated analyses could reflect the results from environmental viromes, we carried out an additional analysis by combining the 1000G simulated virome with the environmental virome generated from NE Atlantic 1.

### Bioinformatic analyses applied to simulated and environmental viromes

Viral sequences and genomes were annotated in simulated and environmental viromes by using different bioinformatic tools. First, such bioinformatic tools were used to assess viral sequences identified in the simulated metagenomes. Then, we evaluated the percentage of correctly annotated viral genomes by each tool. In the simulated viromes containing 1000 genomes (1000G), we also calculated the percentage of correctly identified viral reads and genomes for each viral family (contributing for >1.0% to the assemblage), on the basis of the total number of sequences and genomes belonging to each viral family. Finally, the same bioinformatic tools were used to identify viral sequences and genomes in the environmental viromes.

Sequences were compared to the RefSeq viral genome database (release date: 5^th^ of June, 2015) by means of the BLAST+ program v.2.2.3 suite^30^, locally installed, using the tBLASTx, BLASTn and Megablast algorithms. The web pipelines tested were MG-RAST^26^, MetaVir^22^, VMGAP^28^, NBC^31^ and VIROME^27^. The VMGAP pipeline was used thanks to the support of the J. Craig Venter Institute, whereas the other pipelines are freely available online.

Alignments in MG-RAST were carried out against the RefSeq database for taxonomic analysis. An E-value cutoff of 10^−5^, a minimum identity cutoff of 60% and minimum alignment length of 15 bp were used as parameters for this analysis^26^. The Least Common Ancestor, Best Hits, and Representative Hits algorithms were tested (hereafter defined as LCA, BH and RH).

Within VMGAP, the metagenomic reads were iteratively searched against multiple databases (TIGRFAM, ACLAME, PFAM, nr, CDD and environmental protein databases) with default parameters (coverage > 70%, identity > 30%, E-value cutoff < 10^−10^ and <10^−5^) using translations of all ORFs predicted by MetaGeneAnnotator through a combination of naïve 6-frame translations^45^. Iterative database searches allowed functional assignments of reads through a series of rules to evaluate how informative, reliable and accurate is the result from each search^28^.

MetaVir^22^ allows the taxonomic identification of viral sequences by using the tool GAAS^23^, comparing reads against the RefSeq database and normalizing the results for the genome length of each taxon. Reads were passed through the MetaVir pipeline and searched against the RefSeq database with GAAS, and the taxonomic affiliation of each read was calculated with an E-value cutoff of 10^−5^.

The Naïve Bayesian Classifier tool (here defined NBC^31^) was used to identify viral sequences by comparing their n-mer frequency profiles to those of viral genomes available within the databases provided on its webserver with an n-mer length of 9.

The VIROME pipeline^27^ allows the classification of viral sequences against several different taxonomic and functional (UniRef 100) and environmental (MetaGenomes OnLine) databases; the taxonomic affiliation of reads was calculated with an E-value cutoff of 10^−5^.

In environmental viromes, viral families contributing on average to less than 0.01% to the assemblages were excluded. Contributions of sequences to viral families and the abundance of strains in each family for each environmental sample were then used for cluster analysis (using complete linkage) based on Bray-Curtis similarity using PRIMER-E 6 software.

The evaluation of the corrected identification of genomes from the simulated library combined with environmental viromes was performed by using BLAST’s and MG-RAST algorithms, and MetaVir.

Sequences obtained by our environmental samples were also assembled in order to obtain a comparison with the analysis on the unassembled sequences with the same bioinformatic tools. Assembling was performed with the Newbler software (v. 2.6) with the following parameters: 90% of identity, 40 bp of minimum alignment^46^. After assembling, sequences were quality-trimmed using PRINSEQ^44^ and uploaded to the MetaVir webserver^22^ as contig sequences.

### Data accessibility

Raw DNA sequences obtained by pyrosequencing can be accessed through the iMicrobe portal under the names CAM_SMPL_000835 ("VASVAL242/1"), CAM_SMPL_000842 ("VAGALB1/1"), CAM_SMPL_000843 ("VAWC1/1") and CAM_SMPL_000799 ("Black Sea Sediment Metagenome") within the Moore Marine Phage/Virus Metagenomes project. Viral sequences from NE Atlantic site 2 sample are available on MetaVir under the project EXPLODIVE under the name "Atlantic – Viral."

## Additional Information

**How to cite this article**: Tangherlini, M. *et al*. Assessing viral taxonomic composition in benthic marine ecosystems: reliability and efficiency of different bioinformatic tools for viral metagenomic analyses. *Sci. Rep.* **6**, 28428; doi: 10.1038/srep28428 (2016).

## Supplementary Material

Supplementary Information
