# Supplementary material for: Assessing viral taxonomic composition in benthic marine ecosystems: reliability and efficiency of different bioinformatic tools for viral metagenomic analyses
Source: Sci Rep. 2016 Jun 22;6:28428. doi: 10.1038/srep28428 (PMC4916513; doi:10.1038/srep28428)
Supplement: Supplementary Information [file srep28428-s1.doc]

**Supplementary Information**

**Assessing viral taxonomic composition in benthic marine ecosystems: reliability and efficiency of different bioinformatic tools for viral metagenomic analyses**

**Tangherlini M. ^§^, Dell’Anno A. ^§^, Zeigler Allen L. ^‡^, Riccioni G. ^§^, Corinaldesi C. ^§^**

^§^Department of Environmental and Life Sciences, Polytechnic University of Marche, Via Brecce Bianche, 60131 Ancona, Italy

^‡^ Microbial and Environmental Genomics, J Craig Venter Institute, San Diego, CA, USA

**Supplementary methods**

**Supplementary results**

**Supplementary Figures S1 and S2**

**Supplementary methods**

*Generation of simulated databases for evaluating the NBC efficiency*

To test for the efficiency of NBC in sequence assignment, we generated two additional databases. The first database was composed of 50 random bacterial genomes downloaded from the RefSeq database; the second database was composed of 20 of the bacterial genomes previously downloaded and 20 of the viral genomes used to create the 50G simulated dataset. Then, the NBC software was run on the simulated dataset composed of 50 viral genomes (50G) on both databases (the one comprising 50 random bacterial genomes and the one with both viral and bacterial genomes) with an n-mer length of 9.

**Supplementary results**

*NBC efficiency in sequence assignment*

The analysis carried by using NBC on the 50G dataset and a reference database composed only of bacterial genomes showed that all sequences (100%) were affiliated to a genome within the database. When the same simulated dataset was compared to a reference database composed of bacterial and viral genomes, 88% of the viral sequences were affiliated with the corresponding viral genomes, while the rest was affiliated with bacterial genomes.

**Figure S1.** Number of viral strains correctly identified in the simulated viromes (1000G) and in the simulated viromes combined with an environmental virome (Environmental + Simulated).


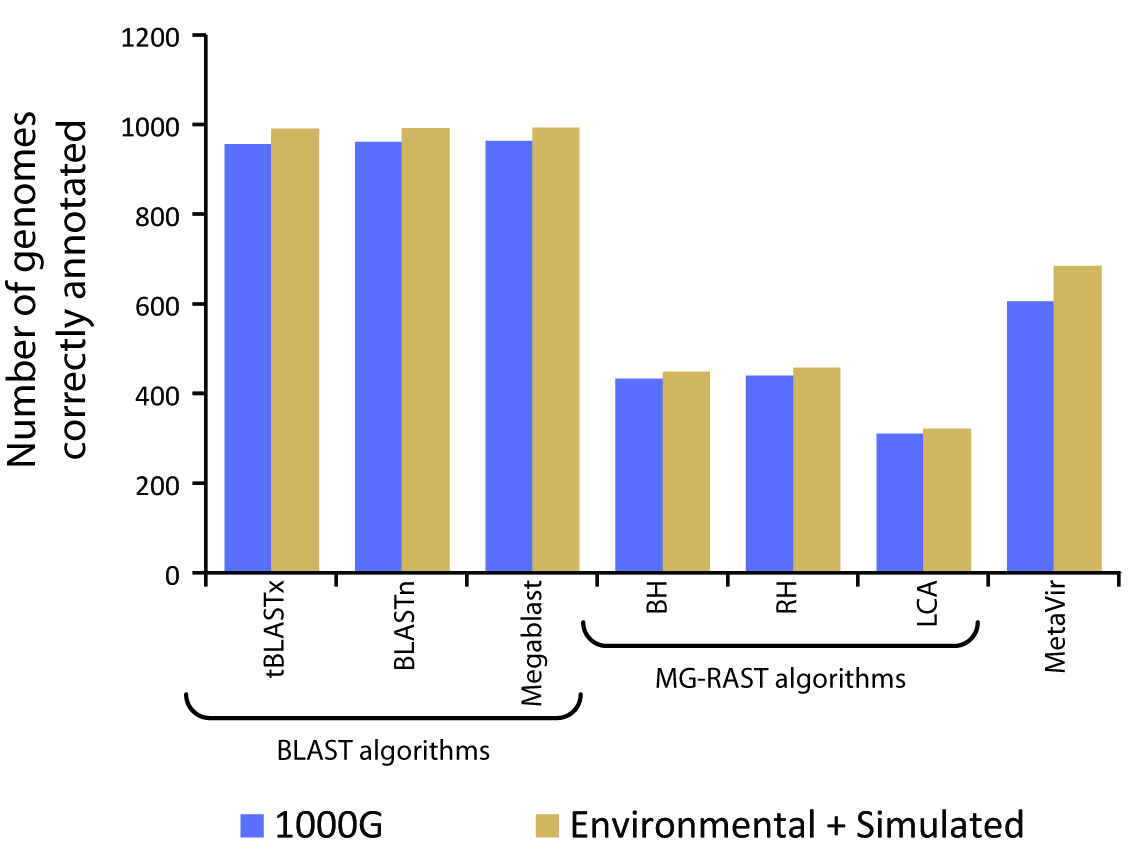


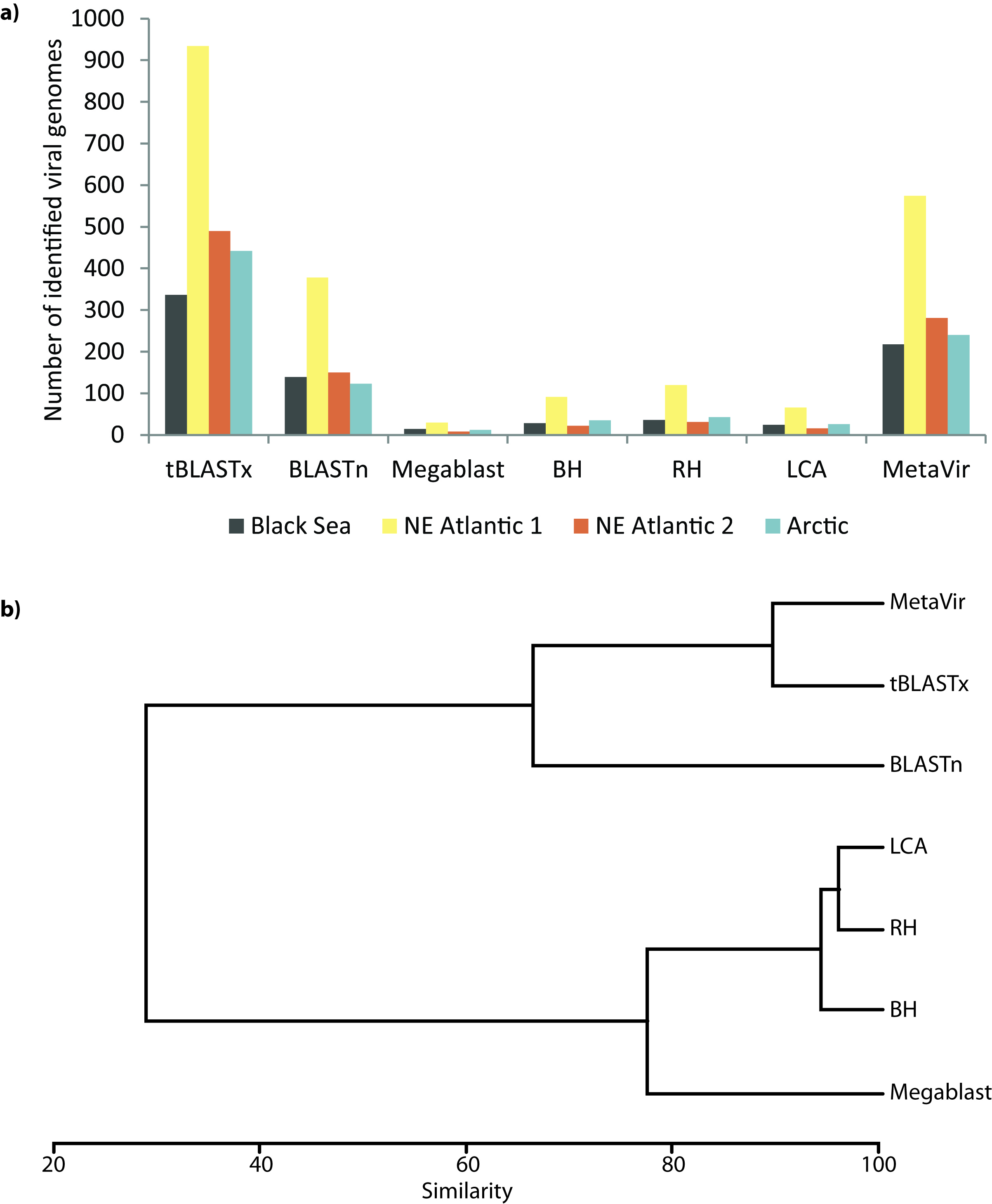
**Figure S2.** A) Number of strains identified by the BLAST and MG-RAST tools and MetaVir after contig assembling. B) Cluster analysis conducted on the viral assemblage composition of environmental viromes (as number of viral strains identified) after contig assembling.
